# The Emerging Role of Hyaluronic Acid as a Multifunctional Regenerative Agent in Periodontal Healing

**DOI:** 10.3390/gels12030205

**Published:** 2026-02-28

**Authors:** Andrei-Mario Bădărău-Șuster, Amelia Tero-Vescan, Mark Slevin

**Affiliations:** 1 Doctoral School of Medicine and Pharmacy, George Emil Palade University of Medicine, Pharmacy, Science, and Technology of Targu Mures, 540139 Targu Mures, Romania; mario.suster@umfst.ro; 2 Department of Oral Rehabilitation and Occlusology, Faculty of Dental Medicine, George Emil Palade University of Medicine, Pharmacy, Science, and Technology of Targu Mures, 38 Gh. Marinescu Str., 540142 Targu Mures, Romania; 3 Biochemistry Department, Faculty of Medicine in English, George Emil Palade University of Medicine, Pharmacy, Science, and Technology of Targu Mures, 540142 Targu Mures, Romania; 4 Center for Advanced Medical and Pharmaceutical Research, George Emil Palade University of Medicine, Pharmacy, Science, and Technology of Targu Mures, 540142 Targu Mures, Romania; mark.slevin@umfst.ro

**Keywords:** hyaluronic acid, hydrogels, periodontitis, periodontal regeneration, bone regeneration, biomaterials

## Abstract

Periodontitis is a multifactorial inflammatory disease characterized by dysbiotic microbial communities and progressive destruction of the supporting periodontal tissues, ultimately leading to alveolar bone loss. Achieving predictable periodontal regeneration remains a major clinical challenge because of the complex interplay between inflammation, microbial burden, and tissue remodeling. In this context, hyaluronic acid (HA), a naturally occurring component of the extracellular matrix (ECM), has gained increasing attention as a bioactive adjunct in periodontal therapy. This narrative review aims to describe current evidence regarding the biological properties, molecular mechanisms, and clinical applications of HA in periodontal therapy, with a particular focus on its immunomodulatory, antimicrobial, and regenerative potential. Available data indicate that HA exerts molecular weight–dependent effects, ranging from anti-inflammatory and extracellular matrix–stabilizing actions to osteogenic and immunostimulatory responses. Clinically, HA has been investigated as an adjunct in both nonsurgical and surgical periodontal therapies, as well as in minimally invasive regenerative approaches, as it has favorable effects on inflammation control, soft tissue healing, and clinical attachment gain. Recent advances in materials science have further expanded the role of HA through the development of engineered hydrogels and hybrid delivery systems incorporating nanoparticles, bioactive glass, growth factors, or antimicrobial agents, which have demonstrated promising osteogenic and antibacterial outcomes in preclinical models. However, the interpretation of existing evidence is limited by heterogeneity in HA formulations, short follow-up periods, and inconsistent reporting of periodontal defect morphology. Future research should focus on standardized, well-designed preclinical and clinical studies integrating histological, radiographic, immunological, and microbiological assessments to distinguish true periodontal regeneration from repair and to optimize HA-based strategies tailored to specific defect configurations.

## 1. Introduction

Periodontitis is a well-recognized, multifactorial inflammatory disease affecting teeth and surrounding tissues and is closely linked to microbial imbalances. Initially, inflammation begins in gingival tissues and, if untreated, progresses to the junctional epithelium, leading to periodontal pocket formation and bone loss. Approximately 90% of the global population is estimated to experience some level of chronic periodontal inflammation, ranging from gingivitis to severe periodontitis. Moreover, several behavioral and systemic factors, including psychological stress, tobacco use, and metabolic disorders such as diabetes mellitus, are well-established contributors to the exacerbation of periodontal inflammation. Consequently, maintaining rigorous personal oral hygiene and attending regular professional dental care appointments remain essential for preserving periodontal health [1]. Over the years, a wide range of therapeutic approaches have been proposed for the management of periodontal disease to control infection, reduce inflammation, and promote the healing of supporting tissues. Mechanical debridement, including professional mechanical plaque removal (PMPR) and SRP, remains the cornerstone of nonsurgical periodontal therapy. Despite the development of various adjunctive methods, SRP continues to be considered the gold standard in periodontal treatment. This procedure effectively disrupts and removes bacterial biofilm and calculus from root surfaces, allowing resolution of inflammation and initial soft tissue reattachment [2].

Tissue healing following periodontal therapy can result in either repair or true regeneration, two biologically distinct processes. Repair typically leads to the formation of a long junctional epithelium along the root surface, restoring continuity but without reestablishing the original attachment apparatus. In contrast, true regeneration refers to the reformation of the alveolar bone, cementum, and periodontal ligament (PDL) that were previously destroyed by disease, thereby restoring the architecture and function of the lost tissues. Achieving complete regeneration remains a significant clinical challenge, largely because of the complex cellular and molecular coordination required among fibroblasts, osteoblasts, endothelial cells, and immune mediators within the periodontal microenvironment [3,4].

In recent years, the use of bioactive molecules and hydrogel-based delivery systems has emerged as a promising strategy to guide the healing process toward regeneration rather than repair. These systems aim to provide a biologically favorable environment by modulating inflammation, enhancing angiogenesis, and stimulating the recruitment and differentiation of progenitor cells [5,6]. Among these agents, hyaluronic acid (HA) has attracted particular attention because of its biocompatibility, biodegradability, and multifunctional biological profile. As a natural component of the extracellular matrix, HA participates in tissue hydration, cell migration, and wound healing. Moreover, depending on its molecular weight, it can exhibit anti-inflammatory, antioxidant, and osteoinductive properties, making it a valuable adjunct in both nonsurgical and regenerative periodontal therapies [7,8,9].

Despite advances in regenerative periodontology, predictable outcomes remain a clinical challenge. Several studies have evaluated HA with other established regenerative materials, such as enamel matrix derivative (EMD), injectable platelet-rich fibrin (I-PRF), and recombinant human fibroblast growth factor-2 (rhFGF-2), and the results have shown promising clinical attachment gains and soft tissue healing [10,11,12]. Among these agents, HA stands out for its dual role in modulating inflammation and promoting tissue regeneration. Its versatile biological profile and proven safety have made it a subject of increasing clinical and scientific interest.

Therefore, this narrative review aims to describe the current evidence concerning the utilization of HA in periodontal therapy, emphasizing its molecular mechanisms, clinical applications, and its potential to act as an adjunctive regenerative agent or as a multifunctional matrix for controlled drug delivery when it is combined with other bioactive regenerative substances in future research directions.

## 2. Hyaluronic Acid—Structure, Properties, and Mechanisms of Action

HA is a naturally occurring, nonsulfated glycosaminoglycan that plays a central role in the structural and functional integrity of connective tissues. It is a linear polysaccharide composed of repeating disaccharide units of D-glucuronic acid and *N*-acetyl-D-glucosamine connected by alternating β-1,3 and β-1,4 glycosidic bonds. Unlike other glycosaminoglycans, which are synthesized in the Golgi apparatus, HA is uniquely produced at the inner surface of the plasma membrane by three specific enzymes, namely, hyaluronan synthases 1, 2, and 3 (HAS1–HAS3), and is secreted directly into the extracellular space. Although HAS1 and HAS2 are generally associated with the synthesis of higher molecular weight hyaluronic acid and HAS3 with shorter chains, HA size is dynamically regulated by UDP-sugar availability, enzyme kinetics, and cellular conditions, leading to considerable overlap in molecular weight distributions [4,13,14].

**Figure 1:**
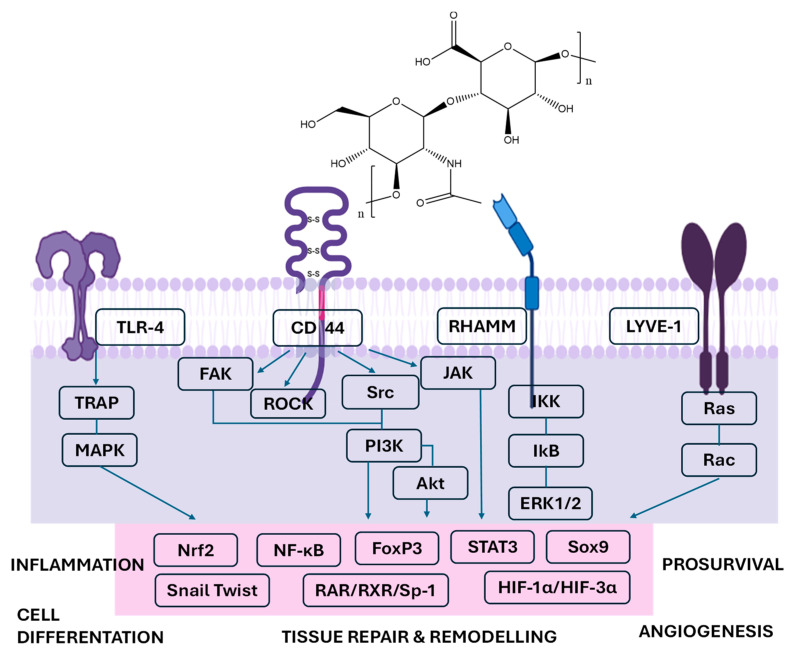
Hyaluronan-mediated signaling pathways involved in inflammation and tissue remodeling (Abbreviations: CD44—cluster of differentiation 44, TLR—toll-like receptor, RHAMM—receptor for hyaluronic acid-mediated motility, LYVE-1—lymphatic vessel endothelial hyaluronan receptor-1, TRAP—TRAF-associated protein, MAPK—mitogen-activated protein kinase, FAK—focal adhesion kinase, ROCK—rho-associated protein kinase, Src—Src family kinases, PI3K—phosphoinositide 3-kinase, Akt—protein kinase B, IKK—IκB kinase, IkB—inhibitor of NF-κB, ERK ½—extracellular signal-regulated kinases ½, RAR—retinoic acid receptor, RXR—retinoid X receptor, Sp-1—specificity protein-1, HIF-1α/HIF-3α—hypoxia-inducible factors, Sox9—SRY-box transcription factor 9, STAT3—signal transducer and activator of transcription 3, FoxP3—forkhead box P3, Nrf2—nuclear factor erythroid 2, NF-κB—nuclear factor kappa-B, Rac = Ras-related C3 botulinum toxin substrate, Ras = rat sarcoma). Image generated with BioRender.com. Hyaluronic acid is a linear glycosaminoglycan composed of repeating disaccharide units of D-glucuronic acid and *N*-acetyl-D-glucosamine.

The structure of HA and the main biological targets are presented in Figure 1.

Chemically, HA is a negatively charged and highly hydrophilic macromolecule, a property conferred by the abundance of carboxyl and hydroxyl groups along its backbone. This structure enables HA to bind substantial amounts of water and contributes to its viscoelastic and lubricating behavior in various connective tissues. Within the extracellular matrix (ECM), HA participates in the organization and stabilization of the periwound environment, where it supports cell migration and the early stages of tissue remodeling. Importantly, clinical and biomolecular evidence indicates that HA application influences specific components involved in wound healing. In a randomized split-mouth clinical trial, Pilloni et al. (2023) reported that adjunctive 0.8% HA gel did not alter early angiogenesis following periodontal surgery, but it significantly enhanced ECM remodeling and collagen maturation, which are the processes considered key drivers of early gingival wound healing and contributors to improved clinical outcomes [15].

In periodontal tissues, HA is naturally present within the gingiva, periodontal ligament, and alveolar bone, where it contributes to the integrity and organization of the extracellular matrix. Rather than acting through a single mechanism, its biological influence depends strongly on molecular weight–dependent signaling. Experimental evidence has shown that HA directly modulates cellular behavior within periodontal tissues. Frasheri et al. demonstrated that different molecular weight fractions exert distinct effects on periodontal ligament cells, with low-molecular-weight HA (LMW-HA) producing the greatest increase in metabolic activity, mineralization, and osteogenic differentiation [16].

At the immunological level, LMW-HA fragments have been shown to activate dendritic cells via Toll-like receptor 4, inducing the release of the cytokines IL-1β, TNF-α, and IL-6, which are known to participate in periodontal tissue breakdown [17]. The biological activity of HA is mediated through specific cell surface receptors, primarily CD44, RHAMM, and TLR-4. Interaction with CD44 promotes fibroblast and osteoblast adhesion, migration, and proliferation, processes crucial for the regeneration of connective and mineralized tissues. Binding to RHAMM influences cytoskeletal reorganization and wound contraction, whereas LMW-HA fragments have been reported to promote inflammatory responses and dendritic cell activation, potentially involving TLR signaling pathways. However, the direct interaction between HA fragments and TLR4 remains debated, as some studies suggest that observed activation may result from indirect mechanisms or experimental contaminants. Through these receptor-mediated pathways, HA acts as a dynamic regulator that orchestrates the transition from inflammation to proliferation, ensuring that healing proceeds in an orderly, biologically balanced manner [18,19,20]. HA has emerged as a promising biomaterial for gene delivery in stem cell based regenerative therapies due to its biocompatibility, biodegradability, and ability to bind CD44 receptors highly expressed on mesenchymal stem cells. HA-based nanoparticles and hydrogel systems enable receptor-mediated cellular uptake and localized delivery of plasmid DNA, siRNA, and microRNA, enhancing transfection efficiency while minimizing cytotoxicity [21]. Chemical modification of HA, including conjugation with cationic polymers or incorporation into composite hydrogels, facilitates nucleic acid complexation and controlled release. Experimental studies demonstrate that HA-mediated gene delivery can promote osteogenic differentiation, enhance angiogenesis, and modulate inflammatory responses in mesenchymal stem cells, supporting tissue regeneration. These properties make HA-based gene delivery platforms particularly attractive for periodontal tissue engineering, where targeted modulation of stem cell behavior is critical for regeneration of bone, periodontal ligament, and connective tissues [22].

Conversely, high-molecular-weight HA (HMW-HA) has a distinct biological profile characterized by stabilizing and anti-inflammatory actions within the periodontal wound microenvironment. Clinical evidence supports these observations. In a randomized clinical trial, Saraç Atagün et al. reported that application of HMW-HA gel (0.2%) compared with standard flossing alone has been associated with improvements in gingival inflammation parameters (GI, PBI), when used as an adjunct to routine oral hygiene measures. These findings implicate a supportive anti-inflammatory effect rather than a replacement for mechanical plaque control [23]. Bo Mi Lee et al. demonstrated that HMW-HA (>1250 kDa), particularly at relatively high concentrations, markedly reduced nitric oxide production in LPS-stimulated macrophages and downregulated key pro-inflammatory mediators, including TNF-α, IL-6, CCL2, and IL-1β. Moreover, in unstimulated macrophages, the same HMW-HA fraction upregulated the expression of anti-inflammatory and prorepair genes, such as IL-10, IL-11, and Arg1, suggesting that the capacity to promote M2 macrophage polarization is independent of exogenous inflammatory stimuli [24].

Taken together, these findings indicate that HMW-HA contributes to periodontal wound healing by suppressing excessive inflammatory signaling, promoting a proresolution immune phenotype, and supporting extracellular matrix stabilization. In contrast with the immunostimulatory and osteoinductive properties of low-molecular-weight HA, these molecular weight–specific activities underscore the dual and context-dependent roles of HA during periodontal tissue repair and regeneration [25].

Another in vitro study comparatively evaluated the biological effects of four different forms of HA, including LMW-HA, oligomeric HA (OHA), HMW-HA, and cross-linked high-molecular-weight HA (CHA). The results demonstrated that both HHA and CHA significantly reduced bacterial counts in newly formed (4 h) biofilms as well as in preexisting five-day-old biofilms. In the absence of biofilm challenge, OHA induced a pro-inflammatory response by increasing IL-1β and IL-10 expression in MONO-MAC-6 cells and increasing IL-8 levels in periodontal ligament fibroblasts (PDLFs) in a time-dependent manner. In contrast, CHA exerted an anti-inflammatory effect by reducing pro-inflammatory cytokines while increasing IL-10 expression in MONO-MAC-6 cells and IL-8 expression in PDLFs. The hyaluronan also reduced IL-1β expression (most markedly with HMW-HA) and increased IL-10 levels in MONO-MAC-6 cells in a molecular weight–dependent manner, with the greatest increase observed for CHA [26].

One of the most distinctive aspects of HA biology is the dynamic balance between its synthesis, degradation, and recycling within periodontal and peri-implant tissues. Rather than acting as a static extracellular matrix component, HA undergoes continuous molecular remodeling, a process that functions as a finely tuned biological switch that coordinates inflammation resolution and subsequent tissue regeneration.

These biological properties, combined with HA’s inherent ability to form hydrogels and interact with other biomolecules, have facilitated its development as a biocompatible carrier for controlled drug delivery and tissue engineering applications. In regenerative periodontology, cross-linked and thermosensitive HA formulations have been specifically engineered to prolong the residence time at the defect site, increase blood clot stability, and sustain the regenerative microenvironment necessary for predictable periodontal repair and regeneration. In a recent study, Zimin Wan et al. developed a pH-responsive hydrogel based on oxidized HA that enabled sustained, localized dual-drug delivery of tobramycin and the antibiotic adjuvant berberine. By adjusting the polymer molecular weight, the hydrogel was tailored for both injectable and dressing applications, while acid-labile imine bonds ensured minimal drug release in healthy tissues and enhanced antibacterial efficacy at infected sites. Overall, the system demonstrated favorable biocompatibility, self-healing behavior, and strong potential as a smart antimicrobial hydrogel for clinical use [27]. These properties suggest that oxidized HA–based, pH-responsive hydrogels may warrant further evaluation as adjunctive platforms in the treatment of periodontitis.

**Figure 2:**
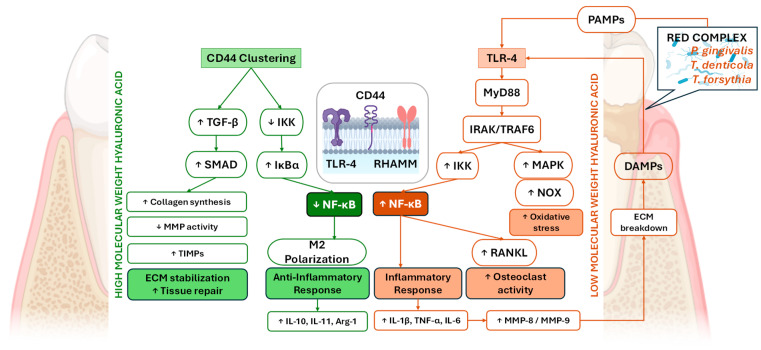
Molecular weight–dependent HA signaling pathways in periodontal disease and tissue repair. HMW-HA exerts protective and pro-regenerative effects on periodontal tissues primarily through CD44 clustering and receptor-mediated signaling. CD44 activation suppresses IKK activity, stabilizes IκBα, and limits NF-κB nuclear translocation, thereby promoting an anti-inflammatory phenotype. This signaling cascade enhances ECM stabilization, increases collagen synthesis, elevates TIMPs, reduces MMP activity, and supports fibroblast migration, angiogenesis, and tissue repair. Additionally, HMW-HA forms a hydrated pericellular matrix that limits DAMP-mediated TLR activation. In contrast, LMW-HA fragments act as DAMPs and activate TLR4-dependent innate immune signaling. TLR4 engagement recruits MyD88 and IRAK kinases, leading to TRAF6/TAK1 mediated activation of both the IKK–NF-κB and MAPK pathways. NF-κB nuclear translocation induces the production of pro-inflammatory cytokines (IL-1β, TNF-α, and IL-6), increases oxidative stress, and upregulates MMP-8 and MMP-9, resulting in ECM degradation. Matrix breakdown further generates additional HA fragments, creating a positive feedback loop that sustains inflammation. Moreover, NF-κB-driven RANKL expression promotes osteoclast differentiation and activity, contributing to alveolar bone resorption and impaired periodontal healing. Together, these opposing mechanisms position HMW-HA as a regulator of tissue homeostasis and repair, whereas LMW-HA amplifies inflammatory signaling and periodontal tissue destruction through NF-κB–dependent pathways. In contrast, periodontal dysbiosis, characterized by pathogenic “red complex” bacteria such as *Porphyromonas gingivalis*, *Treponema denticola*, and *Tannerella forsythia*, results in the release of pathogen-associated molecular patterns (PAMPs), including lipopolysaccharides and fimbriae, which activate TLR2/4. TLR engagement recruits MyD88 and IRAK kinases, leading to TRAF6/TAK1-mediated activation of both the NF-κB and MAPK pathways. (Abbreviations: HA—hyaluronic acid, HMW-HA—high-molecular-weight hyaluronic acid, LMW-HA—low-molecular-weight hyaluronic acid, CD44—cluster of differentiation 44, IKK—IκB kinase, NF-κB—nuclear factor kappa-light-chain-enhancer of activated B cells, ECM—extracellular matrix, TIMPs—tissue inhibitor of metalloproteinases, MMP—matrix metalloproteinase, DAMP—damage-associated molecular pattern, TLR—toll-like receptor, MyD88—myeloid differentiation primary response 88, IRAK—interleukin-1 receptor–associated kinase, TRAF6—TNF receptor–associated factor 6, TAK1—transforming growth factor-β–activated kinase 1, MAPK—mitogen-activated protein kinase, IL-1β/6/10/11—interleukin-1 beta/6/10/11, TNF-α—tumor necrosis factor alpha, RANKL—receptor activator of nuclear factor κB ligand, RHAMM—receptor for hyaluronic acid-mediated motility, PAMPs—pathogen-associated molecular patterns, NOX—NADPH oxidase, DAMPs—damage-associated molecular patterns, Arg-1—Arginase-1, SMAD—Sma + Mad proteins, TGF-β—transforming growth factor beta, IκBα—inhibitor of kappa B alpha, ↓—down arrow means decreased effect/level and ↑—up arrow means increased effect/level).

Although the immunomodulatory effects of HA on cytokine expression play crucial roles in controlling periodontal inflammation, periodontal disease cannot be fully understood without considering the disruption of local homeostasis at the microbial level. Alterations in the composition and balance of the subgingival microbiome lead to dysbiosis, which sustains inflammatory signaling and perpetuates tissue destruction. This imbalance between host defense mechanisms and the subgingival microbial ecosystem represents a key pathogenic driver of periodontal breakdown and recapitulates the importance of therapeutic approaches that aim not only to modulate host cytokine responses but also to restore microbial equilibrium. Molecular weight–dependent HA signaling pathways in periodontal disease and tissue repair connected with the main mechanistic pathways are presented in Figure 2. 

## 3. Considerations of Hyaluronic Acid and Periodontitis from a Microbiological Standpoint

The subgingival microbial ecosystem plays a pivotal role in the initiation and progression of periodontal and peri-implant diseases, with distinct microbial profiles characterizing health and disease. Periodontitis is associated with a dysbiotic biofilm characterized by shifts in microbial community structure and function rather than the overgrowth of individual pathogens alone. While species such as *Porphyromonas gingivalis (Pg)*, *Treponema denticola (Td)*, and *Tannerella forsythia (Tf)*, historically described as the “red complex”, are strongly associated with disease, contemporary models emphasize polymicrobial synergy and dysbiosis (PSD), whereby keystone pathogens disrupt host–microbial homeostasis and promote a pathogenic microbial community. In contrast, periodontal health is associated with a symbiotic microbiota enriched *in genera* such as *Streptococcus* and *Actinomyces*, which contribute to microbial balance and host tissue homeostasis [28,29,30]. Within this context, the modulation of the subgingival microbiota, which involves a shift from red complex–dominated communities toward health-associated bacterial genera, represents a key aspect in the management of periodontitis and peri-implantitis and provides the biological framework for exploring the modulatory effects of HA on the subgingival microbial ecosystem. The use of a 0.2% HA gel as an adjunct to SRP for four consecutive weeks resulted in statistically significant differences between the control and experimental groups at the 6-week follow-up with respect to the gingival index and bleeding on probing (BoP), which favored the test group. Additionally, a significant reduction in the microbial load of red complex pathogens was reported in the experimental group, but the difference was statistically significant only for Tf. These findings suggest that adjunctive HA application may enhance both clinical periodontal outcomes and select microbiological parameters following SRP [31]. Compared with other commonly used antimicrobial agents, such as chlorhexidine (CHX) and azithromycin (AZM), HMW-HA demonstrated a level of bacterial growth inhibition comparable to that of CHX and AZM at their respective minimum inhibitory concentrations (MICs). The minimum inhibitory concentration (MIC) of HA was determined to be 4 mg/mL, whereas substantially lower MIC values were reported for CHX (1.6 µg/mL) and AZM (3.9 µg/mL). The inhibitory effect of HA on Pg growth was similar to that of CHX but inferior to that of AZM [32].

While AZM and CHX are associated with well-documented adverse effects, including antimicrobial resistance development, gastrointestinal disturbances, and dysbiosis to AZM, as well as tooth staining, taste alteration, mucosal irritation, and cytotoxicity, HA has a favorable safety profile characterized by high biocompatibility and minimal reported adverse reactions. HA is generally regarded as biocompatible and safe for living tissues. Nevertheless, mild and transient adverse effects have been reported. These reactions may include localized bruising, swelling, erythema, pain, and pruritus, predominantly at the site of administration, particularly following injectable applications used in gingival augmentation procedures to achieve adequate tissue thickness and an increased amount of keratinized gingival tissue [31,33,34].

Interestingly, HA inhibited Prevotella intermedia and *Fusobacterium nucleatum* in a concentration-dependent manner. *F. nucleatum* exhibited sensitivity at a concentration of 2 mg/mL, whereas *P. intermedia* showed inhibitory sensitivity at a lower concentration of 1 mg/mL, indicating differential susceptibility among periodontal pathogens [35]. The use of a native 0.2% HA gel in combination with SRP for the treatment of periodontitis in both diabetic and nondiabetic individuals resulted in decreases in Pg and Fn levels across almost all groups. Notably, the diabetic HA group presented a nonsignificant increase in the *P. gingivalis* proportion. Although in vitro studies demonstrate inhibitory effects of HA on periodontal pathogens, the clinical application of 0.2% native HA gel has not consistently shown significant additional benefits when used adjunctively to SRP. This discrepancy may be attributed to several factors, including limited subgingival penetration, rapid enzymatic degradation by hyaluronidases, dilution by saliva and gingival crevicular fluid, and the structural complexity and resilience of subgingival biofilms. Furthermore, the dynamic environment of the periodontal pocket may reduce HA bioavailability and retention time, potentially limiting its antimicrobial effectiveness in vivo. The authors noted that native HA has a relatively short half-life (12 h to 3 days) and suggested that cross-linked HA, with improved mechanical properties and longer persistence, may be a more promising candidate for future investigations [36].

## 4. Clinical Applications of Hyaluronic Acid in Periodontal Therapy

As discussed above, HA has demonstrated notable immunological and microbiological benefits in the context of periodontal therapy. Clinically, HA can be applied not only as an adjunct to nonsurgical periodontal treatment but also during the third stage of periodontal therapy in regenerative surgical interventions. Moreover, HA is incorporated as an active component in various oral care products, including mouthwashes and toothpastes, further extending its potential role in maintaining periodontal health and preventing disease progression.

HA exhibits distinctive physicochemical and biological properties that differentiate it from other glycosaminoglycans such as heparin and heparan sulfate, making it particularly suitable for hydrogel formation and periodontal regenerative applications. Unlike sulfated glycosaminoglycans, HA is a nonsulfated, highly hydrophilic polymer capable of retaining large amounts of water, forming viscoelastic hydrogels that mimic the extracellular matrix and support cell migration and tissue repair [37].

Its unique rheological behavior, tunable crosslinking capacity, and biodegradability enable the formation of injectable or scaffold-based hydrogels with controlled degradation and drug release properties. In contrast, heparin and heparan sulfate primarily function as protein-binding cofactors within proteoglycans and are less suited for structural scaffold formation due to their high sulfation and strong anticoagulant activity. Experimental studies demonstrate that HA-based hydrogels enhance periodontal ligament cell proliferation, osteogenic differentiation, and extracellular matrix deposition while modulating inflammation and promoting wound healing. These combined physicochemical and bioactive properties explain why HA-based hydrogels are particularly effective biomaterials for periodontal tissue regeneration and local therapeutic delivery [38].

In recent years, increasing emphasis has been placed on minimally invasive periodontal techniques such as the minimally invasive surgical technique (MIST) and modified MIST (M-MIST). Nevertheless, the elevation of the buccal and oral papillae during flap procedures may still be associated with surgical risks and increased operative time. In this context, a nonsurgical minimally invasive approach, namely, the minimally invasive nonsurgical technique (MINST), has been introduced, providing effective subgingival debridement while minimizing surgical trauma and reducing the risk of gingival recession.

Recent studies have investigated the efficacy of MINST in combination with various adjunctive agents, including EMD, sodium hypochlorite (NaOCl), proanthocyanidins (PACNs), and HA. However, owing to the wide range of HA formulations and their relatively recent application in periodontal therapy, the available evidence remains limited and heterogeneous. A retrospective clinical case series evaluated the adjunctive, flapless application of a sodium hypochlorite–based cleaning gel followed by xHyA during supportive periodontal therapy (SPT) for the nonsurgical management of residual periodontal pockets. At the 6-month re-evaluation, the clinical parameters improved; in this way, the need for further periodontal surgery decreased because this protocol facilitated pocket closure. In addition, bleeding on probing (BoP) was reduced by more than 60%, indicating a substantial improvement in inflammatory status [39].

The adjunctive use of a 0.8% HA gel with MINST did not provide additional benefits in terms of clinical parameters or bone gain, although the results suggested a potential protective effect in limiting post-treatment gingival recession [40]. Similarly, Vincenzo Iorio-Siciliano et al. reported that the treatment of intrabony defects via MINST, with or without xHyA gel, led to statistically significant improvements in clinical outcomes at 3 and 6 months; however, adjunctive xHyA application yielded superior outcomes at 3 months, and these differences were no longer statistically significant at the 6-month follow-up [41].

In a randomized, split-mouth clinical trial evaluating the effect of a 0.8% HA–based gel as an adjunct to surgical periodontal therapy in patients with Stage III periodontitis, intergroup comparisons revealed no statistically significant differences between the test (open flap debridement + HA 0.8%) and control groups (open flap debridement alone) in terms of clinical parameters at baseline, early follow-up, or final evaluation. However, at the intermediate follow-up, the test group demonstrated a statistically significant gain in clinical attachment level (CAL) compared with the control group [42]. Another double-blind, split-mouth clinical trial assessed both clinical outcomes, including CAL gain, probing depth (PD) reduction, and gingival recession (GR), as well as radiographic outcomes, specifically the distance from the cementoenamel junction to the base of the defect (CEJ–BD). Forty intrabony defects were equally allocated to a test group receiving the same 0.8% HA gel in combination with open-flap debridement (OFD) and a control group treated with OFD plus placebo. Over a 12-month follow-up period, the authors concluded that the adjunctive application of HA gel with OFD resulted in superior clinical and radiographic outcomes compared with OFD alone [43].

The adjunctive use of HA in addition to the coronally advanced flap (CAF) technique may contribute to successful root coverage outcomes regarding Miller Class I and Class II gingival recessions, as suggested in a study published in 2022. Nevertheless, the authors emphasized that these findings should be interpreted with caution, as further well-designed studies with larger sample sizes are needed to validate these results and support broader clinical application [44].

A thin gingival phenotype and reduced width of keratinized tissue have been associated with increased susceptibility to soft tissue inflammation and mechanical trauma; however, their direct role in periodontal breakdown remains debated. Evidence suggests that adequate plaque control and supportive maintenance can mitigate potential risks even in sites with limited keratinized tissue. Similarly, reduced keratinized mucosa around implants has been linked to increased plaque accumulation and mucosal inflammation, although a definitive causal relationship with peri-implantitis has not been conclusively established. Traditionally, most approaches aimed at enhancing gingival thickness and keratinized tissue width have relied on surgical interventions. More recently, minimally invasive injectable techniques, including the use of platelet-rich fibrin (I-PRF) to modulate and improve the thin gingival phenotype, have been proposed. Evidence from the literature, which was based on a split-mouth study design, was used to evaluate the effectiveness of both HA and I-PRF in increasing gingival thickness and keratinized tissue width through three consecutive injection sessions performed at one-week intervals. The results demonstrated significant improvements in both parameters at the three-month follow-up from the initial injection, with no statistically significant differences observed between the two biomaterials [34]. Furthermore, achieving an adequate width of keratinized gingiva represents an important goal for maintaining peri-implant tissue health. Reduced keratinized tissue width has been associated with a greater incidence of peri-implantitis, increased plaque accumulation, soft tissue inflammation, mucosal recession, marginal bone loss, and greater patient discomfort, supporting the clinical importance of strategies aimed at enhancing peri-implant soft tissue quality [45].

HA has reparative and regenerative properties. However, other biomaterials, such as platelet-rich fibrin (PRF) and its derivatives, enamel matrix derivatives (EMDs), and membranes used in guided tissue regeneration (GTR), have consistently shown regenerative potential in surgical treatments over time. Manuel Rodríguez compared 1.8% xHyA with EMD in treating one-, two-, and three-wall intrabony defects. No significant differences were found between the groups. Both treatments resulted in significant improvements from baseline at 3, 6, 12, and 18 months. A limitation was that radiographic bone fill was measured only with bidimensional methods. Volumetric analyses (mm^3^) provide a more detailed assessment, but they require invasive techniques and pose accuracy challenges. Larger-scale studies are needed to better understand how defect shape influences outcomes and to compare the benefits of different biomaterials [12].

In a comparable clinical setting, a recent study evaluated the adjunctive use of HA and red I-PRF in combination with PMPR for the nonsurgical treatment of Stage III periodontitis. Patients were allocated to three groups: HA (G1), red I-PRF (G2), and PMPR alone (G3). Compared with PMPR alone, both HA and red I-PRF, when used as adjuncts to PMPR, resulted in significantly greater improvements in periodontal parameters, particularly clinical attachment level gain and probing depth reduction. However, no statistically significant differences were observed between the two biologic adjuncts [46].

In addition to periodontal therapy, HA has also been investigated for minimally invasive soft tissue reconstruction. In the management of interdental papillary loss, HA gel injections, with or without plasma rich in growth factor (PRGF), administered at baseline and during early follow-up, led to measurable improvements in papillary morphology on the basis of image-derived parameters. While HA alone appears effective, the addition of PRGF may provide an adjunctive benefit, suggesting a potential synergistic effect in soft tissue augmentation [47].

Melatonin, a hormone primarily synthesized by the pineal gland and various peripheral tissues, plays a relevant role in multiple physiological processes, including bone remodeling and antioxidant regulation. A clinical study compared the adjunctive use of HA and melatonin in combination with a GTR membrane versus the use of a GTR membrane alone in the treatment of 15 bilateral intrabony defects. The authors reported that the combined application of HA and melatonin resulted in more favorable regenerative outcomes, suggesting a potential synergistic effect in the management of intrabony defects in patients with chronic periodontitis. Nevertheless, the limited sample size and follow-up duration warrant caution, and further studies with longer observation periods and additional microbiological assessments are needed to substantiate these preliminary findings [48].

Taken together, the available evidence suggests that HA may represent a valuable adjunct in both nonsurgical and surgical periodontal therapy. However, the findings across studies remain inconsistent. These discrepancies can be largely attributed to substantial heterogeneity in study design, including variations in follow-up duration, defect morphology and classification, clinical endpoints, and, importantly, the wide range of HA formulations used. Many studies are limited by short-term observation periods, small sample sizes, and incomplete reporting of defect characteristics, which hampers meaningful comparisons and limits the extrapolation of results to routine clinical practice.

Future research should therefore focus on well-designed, adequately powered randomized controlled trials with standardized protocols, including precise defect morphology reporting, uniform clinical and radiographic outcome measures, and longer follow-up periods to assess the stability of clinical improvements over time. Moreover, comparative studies directly evaluating different HA formulations, as well as head-to-head comparisons with established regenerative biomaterials such as PRF, EMD, and GTR membranes, are needed to clarify the specific indications and relative advantages of HA in periodontal regeneration. Finally, the integration of histological, immunological, and microbiological assessments would be essential to distinguish true periodontal regeneration from mere tissue repair and to better elucidate the biological mechanisms underlying HA-mediated clinical effects.

## 5. From Native HA to Engineered Hydrogels

HA is an essential constituent of the extracellular matrix (ECM), together with other glycosaminoglycans, such as heparin, heparan sulfate, chondroitin sulfate, keratan sulfate, and dermatan sulfate. ECM degradation is associated with the activation cascade of matrix metalloproteinases (MMPs), a key feature in the pathogenesis of periodontitis. HMW-HA can be fragmented into LMW-HA through the activity of hyaluronidases (e.g., HYAL1 and HYAL2) and ROS generated during inflammation. These fragments may contribute to pro-inflammatory signaling, whereas MMPs primarily degrade collagen and other extracellular matrix proteins rather than HA.

HA derived hydrogels are three-dimensional hydrophilic polymer networks formed through physical or chemical crosslinking of HA chains, enabling high water retention and viscoelastic behavior that closely mimic the ECM. Their structure can be tailored through crosslinking density, molecular weight, and chemical modification, allowing control over mechanical strength, degradation rate, and drug release kinetics. These hydrogels exhibit excellent biocompatibility, biodegradability, and mucoadhesive properties, making them suitable for localized therapeutic delivery and tissue regeneration. In periodontal applications, HA-based hydrogels provide a hydrated scaffold that supports fibroblast and periodontal ligament cell migration, modulates inflammation, stabilizes the ECM, and enhances wound healing. Their ability to adhere to moist oral tissues and maintain bioactive agents within periodontal pockets further improves therapeutic retention and regenerative outcomes [49].

To overcome this limitation, various chemical modifications, such as methacrylated HA, thiolated HA, or different crosslinking strategies, are currently being investigated to increase HA stability and resistance to enzymatic degradation [50]. A review of the literature revealed that the assessment of MMP-8 levels in gingival crevicular fluid (GCF) represents a rapid and accurate diagnostic approach with significant potential to reduce the failure rate of dental implants by enabling early detection of peri-implant tissue breakdown [51]. Future studies should aim to evaluate the therapeutic response of different HA formulations while also considering local MMP levels in the gingival crevicular fluid to better tailor HA-based interventions in periodontal therapy.

Drug delivery systems can also involve encapsulating pharmaceuticals in carriers such as micelles or nanoparticles, which shield the active compound from degradation and enable targeted delivery to specific body sites. HA and its derivatives are utilized in diverse drug delivery systems, such as cationic polymer gene carriers, gel-based delivery systems, polyelectrolyte microcapsules, films, and nanoparticles [52].

The HA formulations currently used in periodontology generally exhibit relatively low viscosity, raising concerns regarding their medium- and long-term stability within the periodontal pocket, particularly in osseous defects. This limitation is further exacerbated by the hostile environment of the oral cavity, which is constantly subjected to mechanical forces, moisture, and microbial challenges. Methacrylic anhydride (MHA) can be employed to chemically modify HA, enabling UV-induced cross-linking and transforming the HA–based product from a sol into a gel with significantly enhanced stability. Furthermore, such hydrogels can be loaded with mesoporous bioactive glass nanoparticles (MBGNs) and antibacterial agents, such as minocycline hydrochloride (MNCl). As demonstrated in a recent study, an MBGN–MNCl/methacrylated HA gel effectively inhibited the growth of *Streptococcus mutans* and significantly reduced the expression of pro-inflammatory cytokines, including IL-6 and TNF-α. In addition, this composite hydrogel promoted the expression of osteogenesis-related genes, such as ALP, RUNX2, and OPN [53].

Pluronic F127 is composed of poly(ethylene oxide)–poly(propylene oxide) copolymers and is a biocompatible, thermoresponsive material that rapidly transitions from a low-viscosity solution to a solid gel when the temperature exceeds its critical gelation temperature (CGT). Pluronic F127 can be successfully combined with HA, yielding hydrogels that are easy to handle and particularly suitable for nonsurgical periodontal therapy. In addition, spermidine-modified mesoporous polydopamine nanoparticles (PH/M@S) exhibit multiple therapeutic capabilities, including photothermal antibacterial activity, reactive oxygen species (ROS) scavenging, and anti-inflammatory effects, as shown in a rat model of periodontitis. This HA–modified hydrogel incorporating these nanoparticles effectively reduced the bacterial load, alleviated local inflammation, and inhibited alveolar bone resorption [54].

Another thermoresponsive HA–based hydrogel, modified with Pluronic F127 and loaded with bioactive glass (BG) and silver nanoparticles (AgNPs), accelerated the wound healing process by reducing inflammation and bacterial load, while modulating the proliferative phase through molecular mechanisms in infected wounds in a rat model. In summary, Pluronic F127, HA, BG, and AgNPs exhibit synergistic effects in promoting the healing of infected wounds and may have potential for clinical application following further studies [55]. This hydrogel could also be considered for use in an experimental periodontitis model. Alternatively, its efficacy could be evaluated in bone regeneration via critical-size calvarial defect models, providing preclinical insight into its potential for periodontal and alveolar bone regeneration.

HA-based hydrogel microspheres loaded with zinc oxide nanoparticles (ZnO-NPs) fabricated via 3D printing, demonstrated strong antibacterial activity and enhanced osteogenic differentiation of bone marrow stromal cells, as shown by upregulated ALP, OCN, OPN and COL-1 expression. In a rat calvarial defect model, they promoted significant bone regeneration, increased angiogenesis, and elicited an anti-inflammatory response, highlighting their potential as a bone regenerative strategy in infectious conditions [56]. This hydrogel could represent a promising candidate for the treatment of periodontitis, and its efficacy should be further evaluated through histological and radiographic assessments in an experimental periodontitis model.

**Figure 3:**
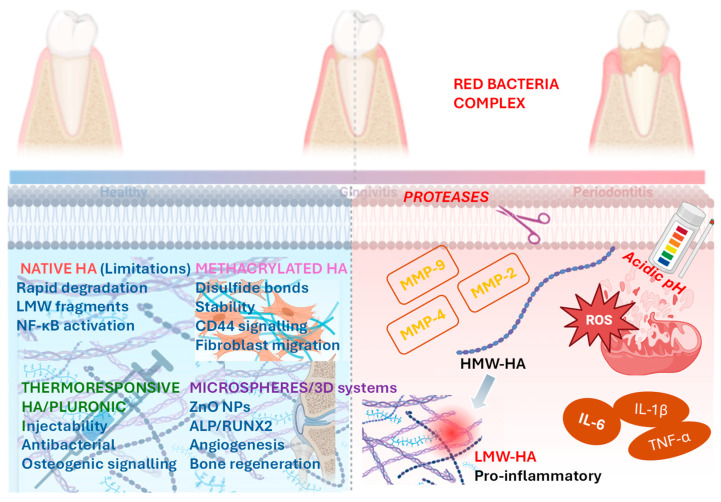
Molecular mechanisms by which advanced hyaluronic acid–based hydrogels modulate inflammation, microbial burden, and regeneration in periodontitis. Schematic overview of the molecular pathways affected by native and engineered HA hydrogels in the periodontal microenvironment. In periodontitis, elevated MMP activity, ROS, and bacterial proteases promote the degradation of HMW-HA into LMW-HA fragments that activate TLR-4/MyD88/NF-κB signaling and sustain pro-inflammatory cytokine production. Chemically modified HA formulations (e.g., methacrylated or cross-linked HA) exhibit increased resistance to enzymatic degradation, suppress inflammatory signaling, and enable the sustained delivery of antibacterial and osteogenic agents. Thermoresponsive HA–Pluronic F127 hydrogels enhance local retention and mechanical stability, whereas HA microspheres and 3D-printed systems loaded with bioactive nanoparticles promote antibacterial activity, angiogenesis, and osteogenic differentiation via the ALP–RUNX2–OPN pathway. Overall, advanced HA hydrogels function as multifunctional platforms that restore the host–microbiome balance, attenuate inflammation, and support periodontal tissue regeneration. (Abbreviations: HA—hyaluronic acid, HMW-HA—high-molecular-weight hyaluronic acid, LMW-HA—low-molecular-weight hyaluronic acid, MMP—matrix metalloproteinase, ROS—reactive oxygen species, TLR-4—toll-like receptor 4, MyD88—myeloid differentiation primary response 88, NF-κB—nuclear factor kappa-B, ALP—Alkaline Phosphatase, RUNX2—runt-related transcription factor 2, OPN—osteopontin, IL-6—interleukin-6, IL-1β—interleukin-1 beta, TNF-α—tumor necrosis factor alpha, CD44—cluster of differentiation 44, ZnO-NP—zinc oxide nanoparticles).

The main molecular mechanisms by which HA hydrogels modulate inflammation, microbial burden, and regeneration in periodontitis are presented in Figure 3. 

Microneedle-based delivery systems have recently emerged as a promising minimally invasive strategy for transmucosal drug delivery in oral tissues. Dissolvable and hydrogel-forming microneedles can penetrate the oral mucosa, enabling localized and sustained release of therapeutic agents while minimizing tissue trauma. In this context, HA is an attractive biomaterial due to its excellent biocompatibility, rapid dissolution, and well-documented anti-inflammatory and regenerative properties in periodontal tissues. A composite dissolvable microneedle patch designed for oral transmucosal administration has demonstrated the practical applicability of this approach. The patch consists of an array of drug-loaded microneedles with HA tip portions and a polyvinylpyrrolidone base, allowing painless penetration of the mucosal barrier and targeted delivery to the basal layer and submucosa. The HA tip enables rapid dissolution and efficient drug release, while a double-layer backing system improves adhesion and reduces saliva washout, enhancing delivery efficiency in the moist oral environment. In vitro and in vivo studies confirmed rapid and efficient drug release within oral mucosa, highlighting the potential of HA-based microneedle systems as a novel strategy for oral mucosal therapy. By improving local bioavailability and enabling controlled delivery, microneedle-assisted hyaluronan administration may represent a promising adjunctive approach in periodontal therapy, promoting tissue regeneration and modulation of local inflammatory responses [57]. Extrusion-based 3D printing technologies are particularly suitable for fabricating hyaluronic acid–containing membranes and scaffolds for periodontal regeneration due to their compatibility with hydrogel biomaterials. In a critical-size periodontal defect canine model, a 3D-printed gelatin/elastin/sodium hyaluronate membrane promoted new bone, periodontal ligament, and cementum formation, with outcomes comparable or superior to commercial collagen membranes, supporting its potential as an alternative biomaterial for guided tissue regeneration [58].

Table 1 provides selected representative examples of HA containing products and highlights their therapeutic applications in periodontal therapy.

## 6. Limitations of Current Evidence and Future Directions

The current evidence is limited by the considerable heterogeneity of hyaluronan-based systems used across studies. Available formulations differ substantially in terms of molecular weight, physicochemical properties, and application protocols, making direct comparisons challenging. Moreover, most studies do not stratify outcomes according to the type and morphology of periodontal defects, which may significantly influence regenerative potential. Another important limitation is represented by the predominantly short- to medium-term follow-up periods reported in the literature. While favorable clinical outcomes have been described, there is insufficient evidence regarding the long-term stability of treated sites. Therefore, future well-designed studies should directly compare different hyaluronan systems and assess the long-term clinical and radiographic stability of periodontal regeneration, ideally in comparison with other established biomaterials.

Most studies evaluating the clinical effects of HA in periodontal therapy focus primarily on clinical parameters, such as probing pocket depth (PPD) reduction and CAL gain. While these outcomes are important, notably, they can result from tissue repair rather than true periodontal regeneration. In contrast, EMD has demonstrated regenerative capacity for more than three decades, with histological evidence clearly showing the formation of new cementum, periodontal ligament, and alveolar bone [63]. This highlights the need for histological studies to substantiate the regenerative potential of different types and concentrations of HA, providing direct evidence of true tissue regeneration rather than mere repair. Expanding such research would be critical to fully understand the biological effects of HA and its potential role in periodontal and peri-implant regenerative therapy.

Studies on regenerative periodontal therapy tend to compare HA with other established biomaterials rather than explore their combined use. This comparative approach, while valuable, limits the understanding of potential synergistic or antagonistic interactions between biomaterials. There is a clear need for additional in vitro, experimental, and subsequently pilot clinical studies designed to evaluate combination therapies and to determine whether these biomaterials act synergistically or interfere with one another.

Evidence from the current literature suggests that the combined application of EMD and HA may exert a synergistic inhibitory effect on lipopolysaccharide (LPS)-induced inflammatory responses, achieved through a reduction in pro-inflammatory cytokine expression and the subsequent enhancement of wound healing and cell migration. Notably, the combination of EMD and HA has been shown to significantly downregulate the expression of early pro-inflammatory cytokines. Nevertheless, further animal studies and advanced three-dimensional in vitro models are needed to elucidate the regenerative potential of such combinations at the level of cell–biomaterial interactions before their routine clinical application can be considered [64].

Despite the growing body of evidence supporting the adjunctive use of HA in periodontal therapy, substantial gaps remain in the available literature. Considerable inconsistency exists in study designs, outcome measures, and treatment protocols, making direct comparisons challenging. Follow-up durations vary widely, with many studies limited to short- or medium-term evaluations, thereby restricting conclusions regarding the long-term stability of clinical and radiographic outcomes. In addition, defect morphology, particularly wall number, defect type, and depth, is often insufficiently reported or inconsistently classified, despite its known influence on regenerative potential. Finally, marked heterogeneity in HA formulations, including differences in concentration, molecular weight, cross-linking methods, and delivery systems, further complicates the interpretation of results and limits the ability to draw robust, generalized conclusions.

Periodontal disease primarily presents two bone resorption patterns: vertical and horizontal defects. Vertical defects are categorized on the basis of the number of remaining osseous walls, such as three-wall, two-wall, and one-wall defects. When assessing the effectiveness of a new biomaterial, it is crucial to link the treatment response to the specific bone defect type, as outcomes can differ. Therefore, choosing the right biomaterial and therapeutic strategy should depend on the shape of the defect, since regenerative potential and clinical results vary with defect configuration.

## 7. Conclusions

Current evidence supports the role of HA as a versatile and biologically active adjunct in periodontal therapy, primarily through its anti-inflammatory, wound-healing, and host-modulatory effects. While HA may exert indirect antimicrobial influences by altering the inflammatory microenvironment and biofilm ecology, its clinical benefits appear to be driven mainly by its regenerative and matrix-stabilizing properties. The heterogeneity of HA-based products, variability in follow-up duration, and inconsistent reporting of defect configurations limit direct comparisons between studies and preclude definitive conclusions regarding true periodontal regeneration. Future research should prioritize well-designed preclinical and clinical studies that integrate standardized defect classification, long-term histological and radiographic evaluation, and immunological assessments to better elucidate the regenerative mechanisms of HA.

## Figures and Tables

**Table 1:** HA–based formulations in periodontal regeneration.

Application	HA Formulation	Study Design	Main Outcomes	Ref.
Subgingival adjunct to SRP in chronic periodontitis	0.2% HA gel (Gengigel^®^)	Clinical + histological study	Significant improvement in gingival and bleeding indices; reduced inflammatory infiltrate histologically; no additional improvement in PPD or attachment levels	[59]
Topical application adjunct to oral hygiene in plaque-induced gingivitis	0.2% HA gel (Gengigel^®^)	Randomized, placebo-controlled clinical trial	Significant improvement in Gingival Index and Papilla Bleeds; no significant plaque reduction; shift toward healthier microbiota without significant intergroup microbiological differences	[60]
Stem cell delivery & tissue engineering	Dual-cross-linked oxidized HA–chitosan injectable hydrogel	Experimental biomaterial study	Rapid gelation, high injectability, improved mechanical stability, ~92% stem cell viability after extrusion	[61]
LMW vs. HMW HA	HA formulation: Native hyaluronic acid solutions (HMW, MMW, LMW, ULMW)	In vitro	Increased osteogenic differentiation and mineralization	[16]
HMW-HA and cross-linked HA in periodontal biofilm and immune cell modulation	HMW-HA, LMW-HA, oligomeric HA, cross-linked HA	In vitro biofilm–immune interaction study	HMW-HA and cross-linked HA reduced biofilm counts; decreased IL-1β and ROS; increased IL-10; oligomeric HA induced pro-inflammatory responses	[26]
Ultra-fine 3D bioprinting & disease modeling	Dynamic disulfide-cross-linked HA bioink (cysteine-modified HA + KI)	Experimental bioprinting study	Accelerated gelation, high print fidelity, radical scavenging properties, high cell viability, and support for complex tissue constructs	[62]
Injectable Pluronic F127–HAMA hydrogel as periodontal pocket filling material	Injectable Pluronic F127–HAMA hydrogel with spermidine-modified mesoporous polydopamine nanoparticles	In vitro + rat periodontitis model	Photothermal antibacterial activity, ROS scavenging, anti-inflammatory effects (ERK1/2, NF-κB), restored osteogenesis, reduced bone loss	[54]

Abbreviations: HA—hyaluronic acid, HMW—high molecular weight, MMW—medium molecular weight, LMW—low molecular weight, ULMW—ultra-low molecular weight, SRP—scaling and root planning, PPD—probing pocket depth, ROS—reactive oxygen species, ERK1/2—extracellular signal-regulated kinase ½, NF-κB—nuclear factor kappa B, KI—potassium iodide, HAMA—hyaluronic acid methacrylate.

## Data Availability

No new data were created or analyzed in this study. Data sharing is not applicable to this article.

## Abbreviations

The following abbreviations are used in this manuscript:

HA	Hyaluronic Acid
ECM	Extracellular Matrix
HMW-HA	High-Molecular-Weight Hyaluronic Acid
LMW-HA	Low-Molecular-Weight Hyaluronic Acid
CHA	Cross-Linked High-Molecular-Weight Hyaluronic Acid
SRP	Scaling and Root Planing
PMPR	Professional Mechanical Plaque Removal
PPD	Probing Pocket Depth
CAL	Clinical Attachment Level
BoP	Bleeding on Probing
MIST	Minimally Invasive Surgical Technique
M-MIST	Modified Minimally Invasive Surgical Technique
MINST	Minimally Invasive Nonsurgical Technique
SPT	Supportive Periodontal Therapy
OFD	Open Flap Debridement
CAF	Coronally Advanced Flap
EMD	Enamel Matrix Derivative
PRF	Platelet-Rich Fibrin
I-PRF	Injectable Platelet-Rich Fibrin
PRGF	Plasma Rich in Growth Factors
GTR	Guided Tissue Regeneration
GCF	Gingival Crevicular Fluid
IL	Interleukin
TNF-α	Tumor Necrosis Factor Alpha
ROS	Reactive Oxygen Species
ALP	Alkaline Phosphatase
RUNX2	Runt-Related Transcription Factor 2
OPN	Osteopontin
OCN	Osteocalcin
COL-1	Type I Collagen
MHA	Methacrylated Hyaluronic Acid
xHyA	Cross-Linked Hyaluronic Acid
CGT	Critical Gelation Temperature
MBGN	Mesoporous Bioactive Glass Nanoparticles
BG	Bioactive Glass
AgNPs	Silver Nanoparticles
ZnO-NPs	Zinc Oxide Nanoparticles
